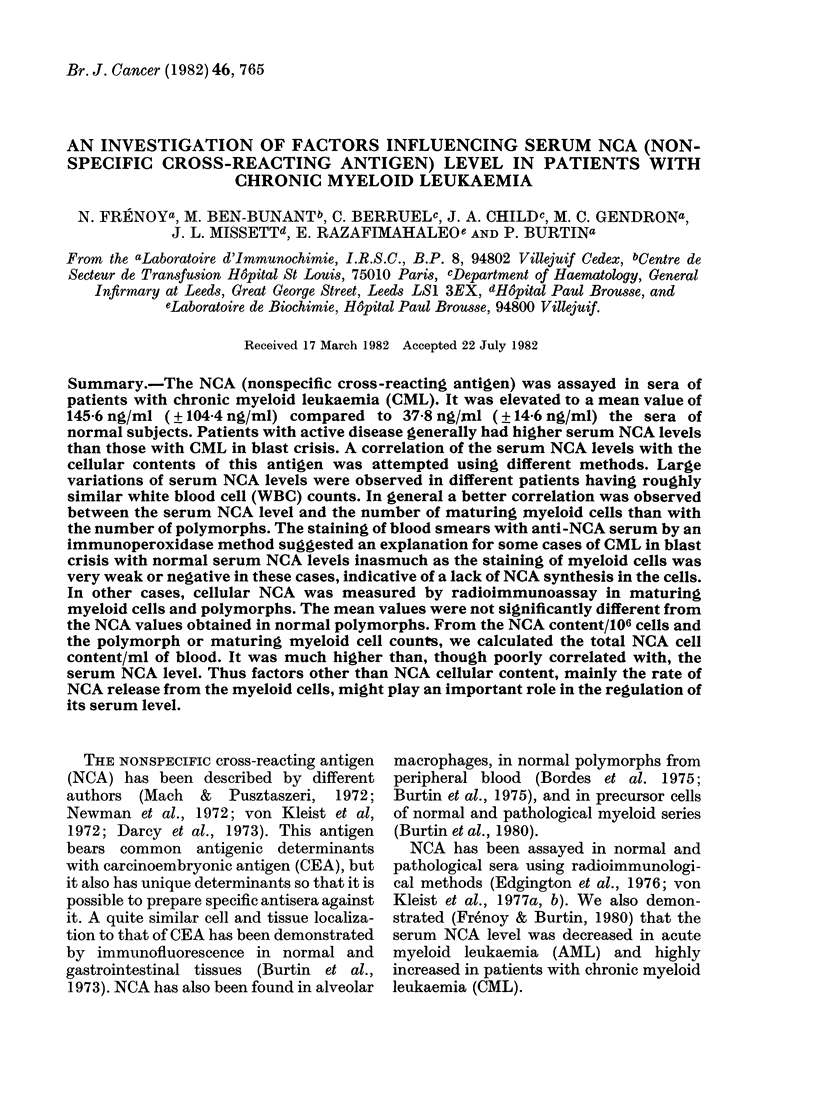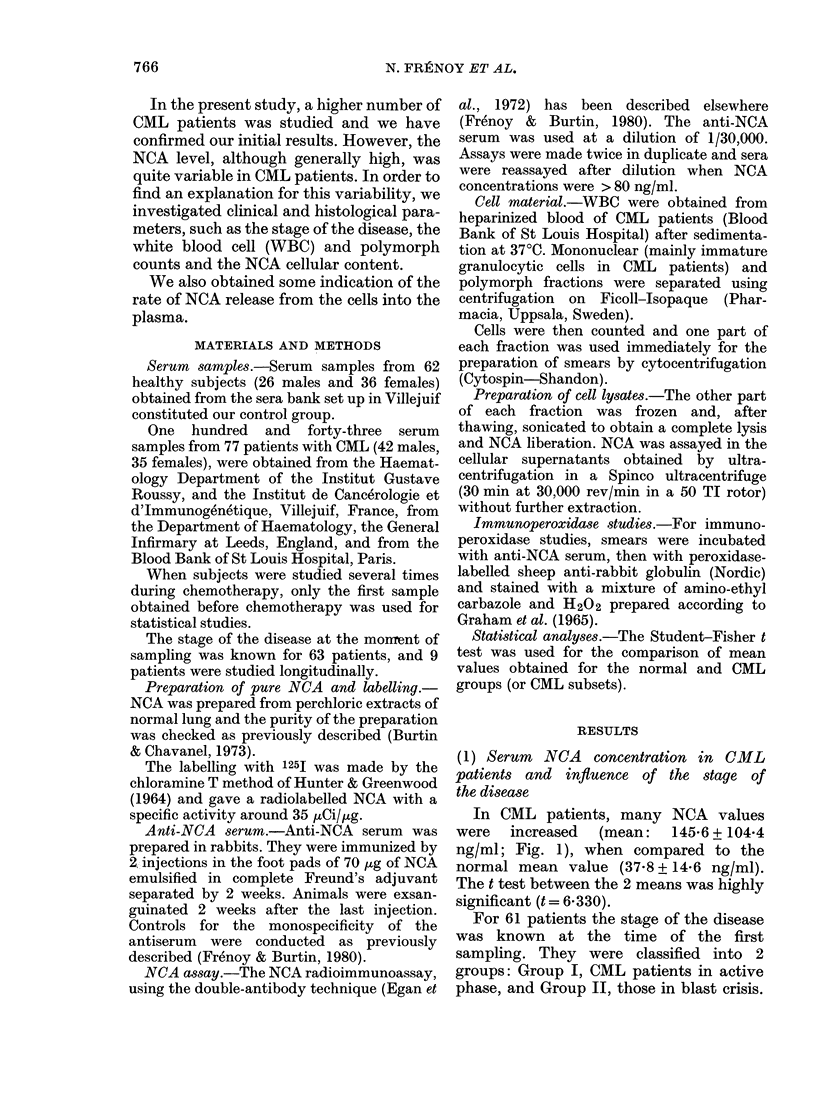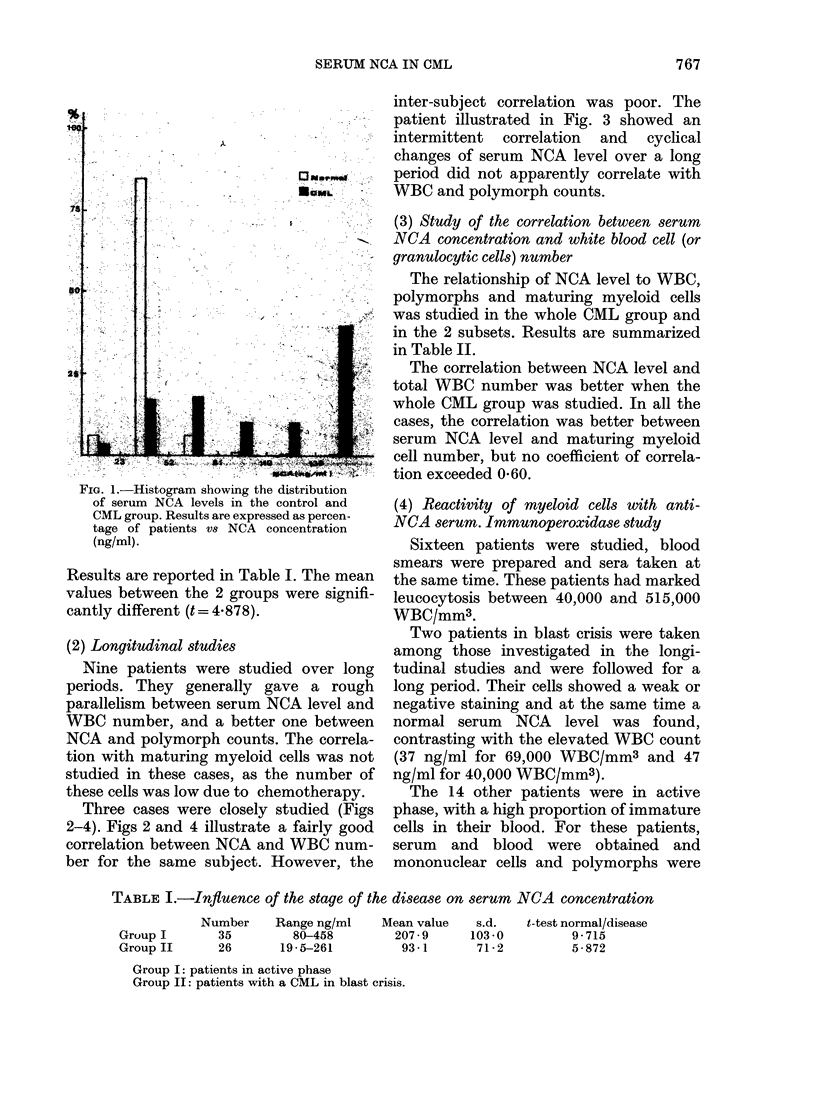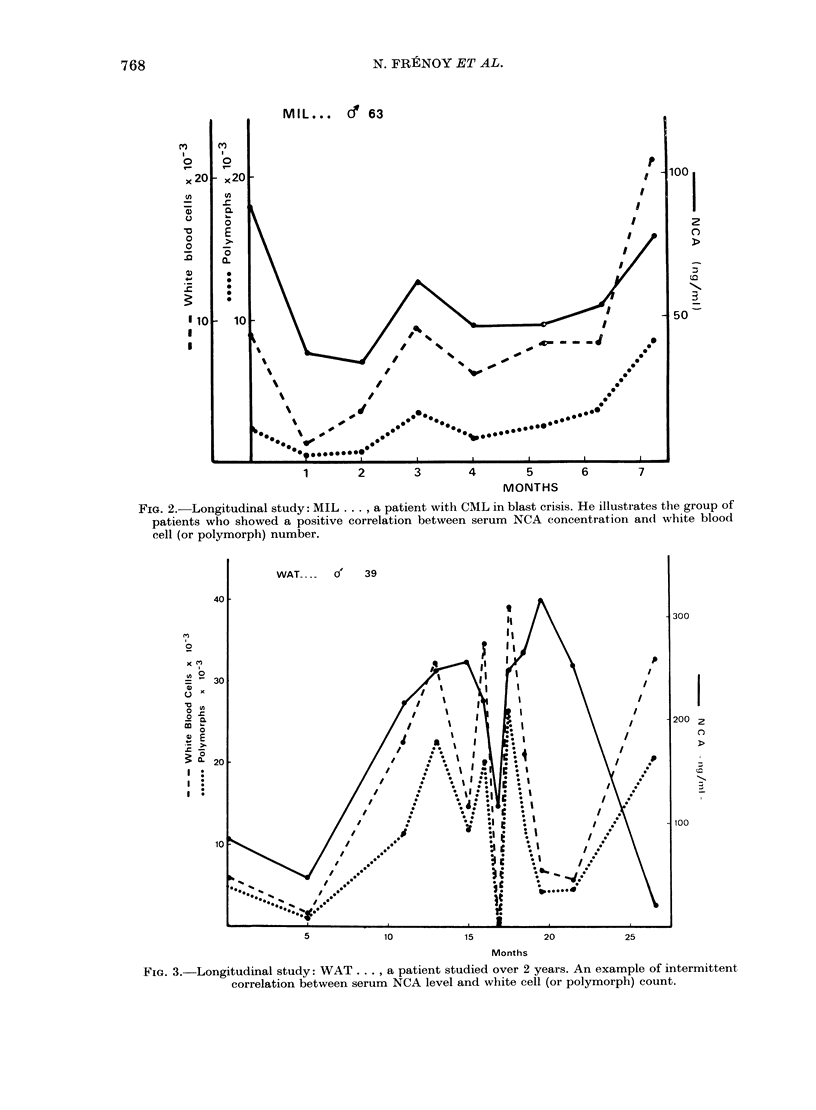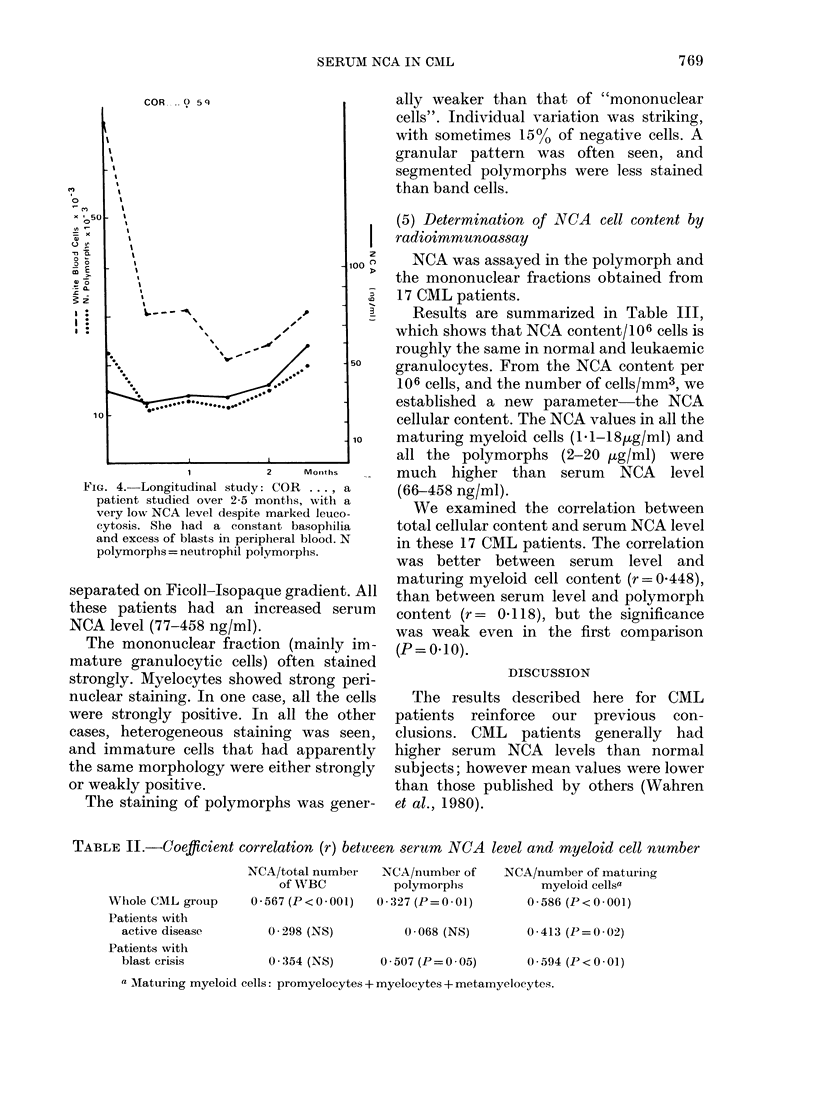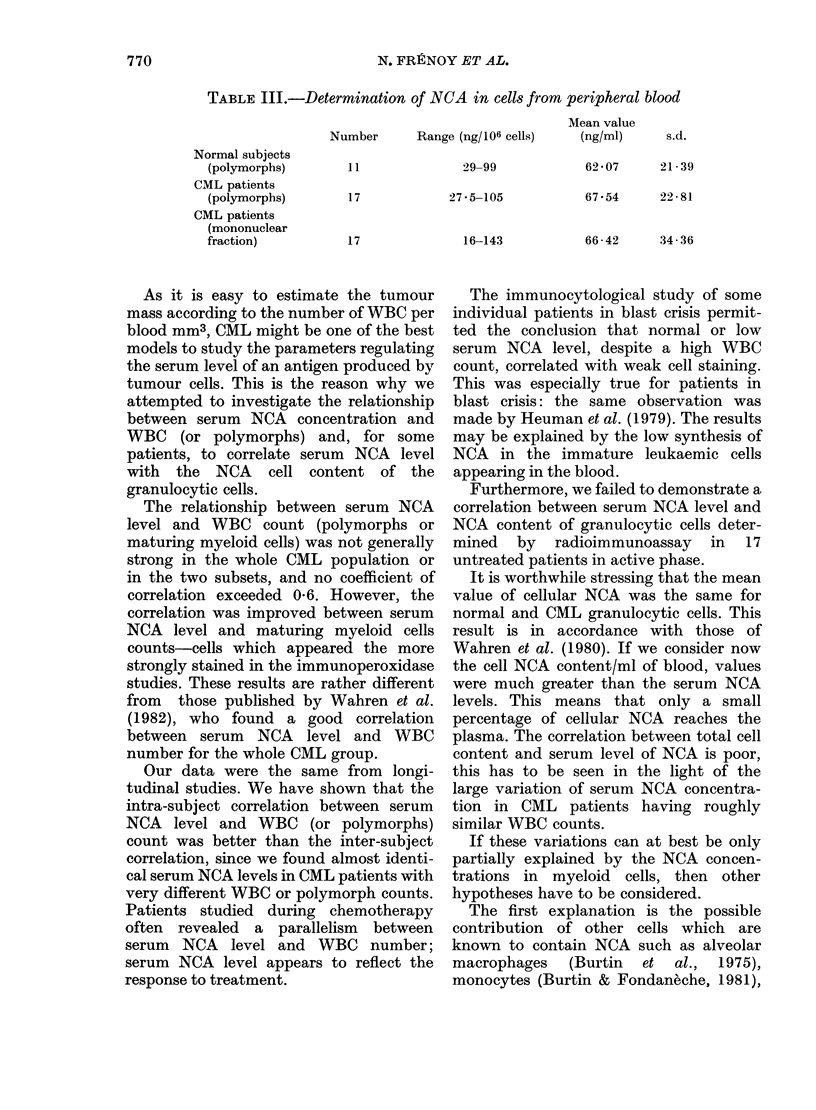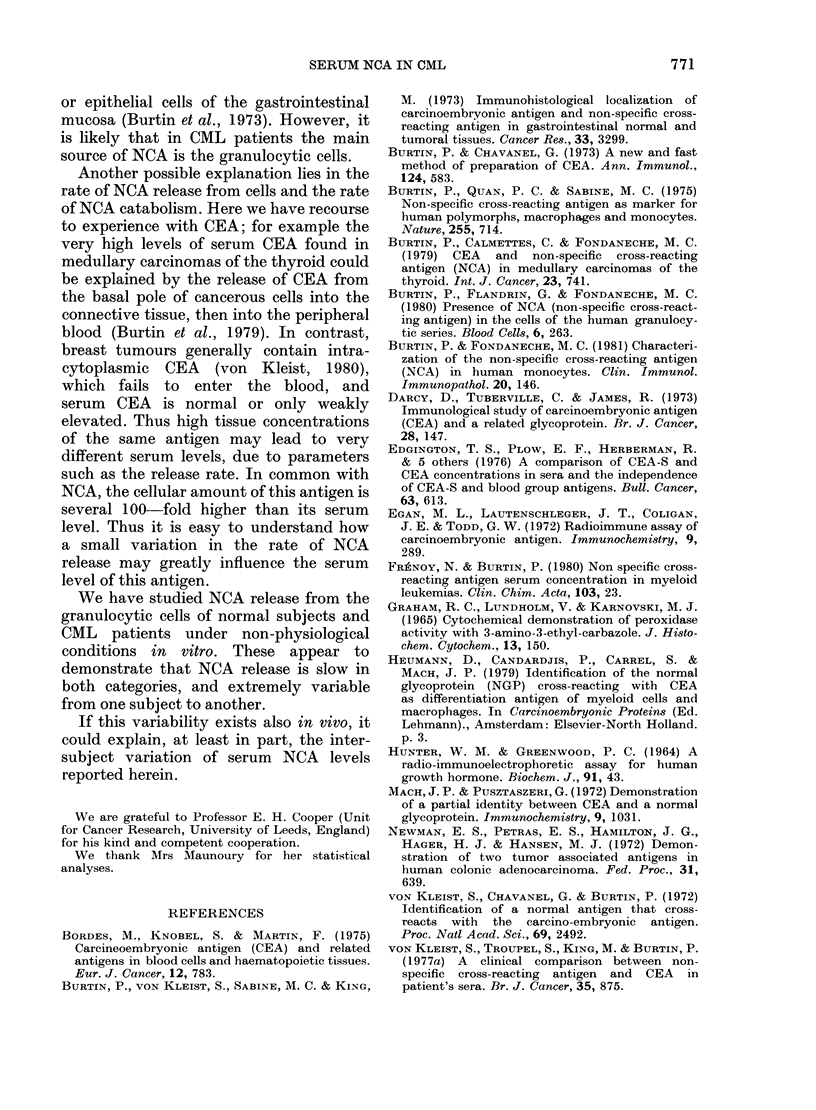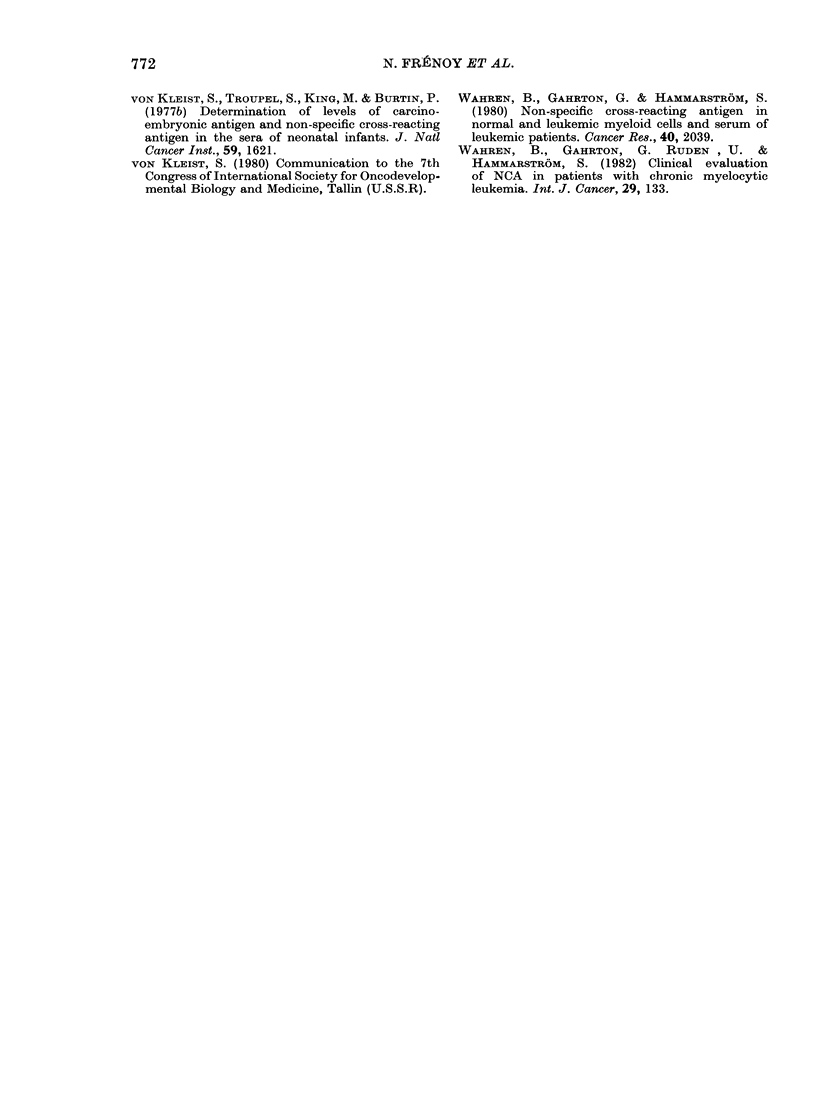# An investigation of factors influencing serum NCA (non-specific cross-reacting antigen) level in patients with chronic myeloid leukaemia

**DOI:** 10.1038/bjc.1982.269

**Published:** 1982-11

**Authors:** N. Frénoy, M. Ben-Bunant, C. Berruel, J. A. Child, M. C. Gendron, J. L. Missett, E. Razafimahaleo, P. Burtin

## Abstract

The NCA (nonspecific cross-reacting antigen) was assayed in sera of patients with chronic myeloid leukaemia (CML). It was elevated to a mean value of 145·6 ng/ml (± 104·4 ng/ml) compared to 37·8 ng/ml (± 14·6 ng/ml) the sera of normal subjects. Patients with active disease generally had higher serum NCA levels than those with CML in blast crisis. A correlation of the serum NCA levels with the cellular contents of this antigen was attempted using different methods. Large variations of serum NCA levels were observed in different patients having roughly similar white blood cell (WBC) counts. In general a better correlation was observed between the serum NCA level and the number of maturing myeloid cells than with the number of polymorphs. The staining of blood smears with anti-NCA serum by an immunoperoxidase method suggested an explanation for some cases of CML in blast crisis with normal serum NCA levels inasmuch as the staining of myeloid cells was very weak or negative in these cases, indicative of a lack of NCA synthesis in the cells. In other cases, cellular NCA was measured by radioimmunoassay in maturing myeloid cells and polymorphs. The mean values were not significantly different from the NCA values obtained in normal polymorphs. From the NCA content/10^6^ cells and the polymorph or maturing myeloid cell counts, we calculated the total NCA cell content/ml of blood. It was much higher than, though poorly correlated with, the serum NCA level. Thus factors other than NCA cellular content, mainly the rate of NCA release from the myeloid cells, might play an important role in the regulation of its serum level.


*Br. J. Cancer* (1982) **46**, 765

### AN INVESTIGATION OF FACTORS INFLUENCING SERUM NCA (NON-SPECIFIC CROSS-REACTING ANTIGEN) LEVEL IN PATIENTS WITH CHRONIC MYELOID LEUKAEMIA

N. FRÉNOY[a], M. BEN-BUNANT[b], C. BERRUEL[c], J. A. CHILD[c], M. C. GENDRON[a], J. L. MISSETT[d], E. RAZAFIMAHALEO[e] AND P. BURTIN[a]

*From the [a]Laboratoire d'Immunochimie, I.R.S.C., B.P. 8, 94802 Villejuif Cedex, [b]Centre de Secteur de Transfusion Hôpital St Louis, 75010 Paris, [c]Department of Haematology, General Infirmary at Leeds, Great George Street, Leeds LS1 3EX, [d]Hôpital Paul Brousse, and [e]Laboratoire de Biochimie, Hôpital Paul Brousse, 94800 Villejuif.*

Received 17 March 1982 Accepted 22 July 1982

**Summary.—The NCA (nonspecific cross-reacting antigen) was assayed in sera of patients with chronic myeloid leukaemia (CML). It was elevated to a mean value of 145·6 ng/ml (±104·4 ng/ml) compared to 37·8 ng/ml (±14·6 ng/ml) the sera of normal subjects. Patients with active disease generally had higher serum NCA levels than those with CML in blast crisis. A correlation of the serum NCA levels with the cellular contents of this antigen was attempted using different methods. Large variations of serum NCA levels were observed in different patients having roughly similar white blood cell (WBC) counts. In general a better correlation was observed between the serum NCA level and the number of maturing myeloid cells than with the number of polymorphs. The staining of blood smears with anti-NCA serum by an immunoperoxidase method suggested an explanation for some cases of CML in blast crisis with normal serum NCA levels inasmuch as the staining of myeloid cells was very weak or negative in these cases, indicative of a lack of NCA synthesis in the cells. In other cases, cellular NCA was measured by radioimmunoassay in maturing myeloid cells and polymorphs. The mean values were not significantly different from the NCA values obtained in normal polymorphs. From the NCA content/10$^{6}$ cells and the polymorph or maturing myeloid cell counts, we calculated the total NCA cell content/ml of blood. It was much higher than, though poorly correlated with, the serum NCA level. Thus factors other than NCA cellular content, mainly the rate of NCA release from the myeloid cells, might play an important role in the regulation of its serum level.**

THE NONSPECIFIC cross-reacting antigen (NCA) has been described by different authors (Mach & Pusztaszeri, 1972; Newman *et al.*, 1972; von Kleist *et al*, 1972; Darcy *et al.*, 1973). This antigen bears common antigenic determinants with carcinoembryonic antigen (CEA), but it also has unique determinants so that it is possible to prepare specific antisera against it. A quite similar cell and tissue localization to that of CEA has been demonstrated by immunofluorescence in normal and gastrointestinal tissues (Burtin *et al.*, 1973). NCA has also been found in alveolar macrophages, in normal polymorphs from peripheral blood (Bordes *et al.* 1975; Burtin *et al.*, 1975), and in precursor cells of normal and pathological myeloid series (Burtin *et al.*, 1980).

NCA has been assayed in normal and pathological sera using radioimmunological methods (Edgington *et al.*, 1976; von Kleist *et al.*, 1977*a*, *b*). We also demonstrated (Frénoy & Burtin, 1980) that the serum NCA level was decreased in acute myeloid leukaemia (AML) and highly increased in patients with chronic myeloid leukaemia (CML).

In the present study, a higher number of CML patients was studied and we have confirmed our initial results. However, the NCA level, although generally high, was quite variable in CML patients. In order to find an explanation for this variability, we investigated clinical and histological parameters, such as the stage of the disease, the white blood cell (WBC) and polymorph counts and the NCA cellular content.

We also obtained some indication of the rate of NCA release from the cells into the plasma.

### MATERIALS AND METHODS

*Serum samples.*—Serum samples from 62 healthy subjects (26 males and 36 females) obtained from the sera bank set up in Villejuif constituted our control group.

One hundred and forty-three serum samples from 77 patients with CML (42 males, 35 females), were obtained from the Haematology Department of the Institut Gustave Roussy, and the Institut de Cancérologie et d'Immunogénétique, Villejuif, France, from the Department of Haematology, the General Infirmary at Leeds, England, and from the Blood Bank of St Louis Hospital, Paris.

When subjects were studied several times during chemotherapy, only the first sample obtained before chemotherapy was used for statistical studies.

The stage of the disease at the moment of sampling was known for 63 patients, and 9 patients were studied longitudinally.

*Preparation of pure NCA and labelling.*—NCA was prepared from perchloric extracts of normal lung and the purity of the preparation was checked as previously described (Burtin & Chavanel, 1973).

The labelling with $^{125}I$ was made by the chloramine T method of Hunter & Greenwood (1964) and gave a radiolabelled NCA with a specific activity around 35 $\mu$Ci/$\mu$g.

*Anti-NCA serum.*—Anti-NCA serum was prepared in rabbits. They were immunized by 2 injections in the foot pads of 70 $\mu$g of NCA emulsified in complete Freund's adjuvant separated by 2 weeks. Animals were exsanguinated 2 weeks after the last injection. Controls for the monospecificity of the antiserum were conducted as previously described (Frénoy & Burtin, 1980).

*NCA assay.*—The NCA radioimmunoassay, using the double-antibody technique (Egan *et al.*, 1972) has been described elsewhere (Frénoy & Burtin, 1980). The anti-NCA serum was used at a dilution of 1/30,000. Assays were made twice in duplicate and sera were reassayed after dilution when NCA concentrations were > 80 ng/ml.

*Cell material.*—WBC were obtained from heparinized blood of CML patients (Blood Bank of St Louis Hospital) after sedimentation at 37°C. Mononuclear (mainly immature granulocytic cells in CML patients) and polymorph fractions were separated using centrifugation on Ficoll–Isopaque (Pharmacia, Uppsala, Sweden).

Cells were then counted and one part of each fraction was used immediately for the preparation of smears by cytocentrifugation (Cytospin—Shandon).

*Preparation of cell lysates.*—The other part of each fraction was frozen and, after thawing, sonicated to obtain a complete lysis and NCA liberation. NCA was assayed in the cellular supernatants obtained by ultracentrifugation in a Spinco ultracentrifuge (30 min at 30,000 rev/min in a 50 TI rotor) without further extraction.

*Immunoperoxidase studies.*—For immunoperoxidase studies, smears were incubated with anti-NCA serum, then with peroxidase-labelled sheep anti-rabbit globulin (Nordic) and stained with a mixture of amino-ethyl carbazole and $H_2O_2$ prepared according to Graham *et al.* (1965).

*Statistical analyses.*—The Student–Fisher $t$ test was used for the comparison of mean values obtained for the normal and CML groups (or CML subsets).

### RESULTS

### (1) *Serum NCA concentration in CML patients and influence of the stage of the disease*

In CML patients, many NCA values were increased (mean: $145{\cdot}6 \pm 104{\cdot}4$ ng/ml; Fig. 1), when compared to the normal mean value ($37{\cdot}8 \pm 14{\cdot}6$ ng/ml). The $t$ test between the 2 means was highly significant ($t = 6{\cdot}330$).

For 61 patients the stage of the disease was known at the time of the first sampling. They were classified into 2 groups: Group I, CML patients in active phase, and Group II, those in blast crisis.

FIG. 1.—Histogram showing the distribution of serum NCA levels in the control and CML group. Results are expressed as percentage of patients *vs* NCA concentration (ng/ml).

Results are reported in Table I. The mean values between the 2 groups were significantly different ($t = 4 \cdot 878$).

(2) *Longitudinal studies*

Nine patients were studied over long periods. They generally gave a rough parallelism between serum NCA level and WBC number, and a better one between NCA and polymorph counts. The correlation with maturing myeloid cells was not studied in these cases, as the number of these cells was low due to chemotherapy.

Three cases were closely studied (Figs 2–4). Figs 2 and 4 illustrate a fairly good correlation between NCA and WBC number for the same subject. However, the inter-subject correlation was poor. The patient illustrated in Fig. 3 showed an intermittent correlation and cyclical changes of serum NCA level over a long period did not apparently correlate with WBC and polymorph counts.

(3) *Study of the correlation between serum NCA concentration and white blood cell (or granulocytic cells) number*

The relationship of NCA level to WBC, polymorphs and maturing myeloid cells was studied in the whole CML group and in the 2 subsets. Results are summarized in Table II.

The correlation between NCA level and total WBC number was better when the whole CML group was studied. In all the cases, the correlation was better between serum NCA level and maturing myeloid cell number, but no coefficient of correlation exceeded 0·60.

(4) *Reactivity of myeloid cells with anti-NCA serum. Immunoperoxidase study*

Sixteen patients were studied, blood smears were prepared and sera taken at the same time. These patients had marked leucocytosis between 40,000 and 515,000 WBC/mm$^3$.

Two patients in blast crisis were taken among those investigated in the longitudinal studies and were followed for a long period. Their cells showed a weak or negative staining and at the same time a normal serum NCA level was found, contrasting with the elevated WBC count (37 ng/ml for 69,000 WBC/mm$^3$ and 47 ng/ml for 40,000 WBC/mm$^3$).

The 14 other patients were in active phase, with a high proportion of immature cells in their blood. For these patients, serum and blood were obtained and mononuclear cells and polymorphs were

TABLE I.—*Influence of the stage of the disease on serum NCA concentration*

| | Number | Range ng/ml | Mean value | s.d. | $t$-test normal/disease |
|---|---|---|---|---|---|
| Group I | 35 | 80–458 | 207·9 | 103·0 | 9·715 |
| Group II | 26 | 19·5–261 | 93·1 | 71·2 | 5·872 |

Group I: patients in active phase
Group II: patients with a CML in blast crisis.

FIG. 2.—Longitudinal study: MIL . . . , a patient with CML in blast crisis. He illustrates the group of patients who showed a positive correlation between serum NCA concentration and white blood cell (or polymorph) number.

FIG. 3.—Longitudinal study: WAT . . . , a patient studied over 2 years. An example of intermittent correlation between serum NCA level and white cell (or polymorph) count.

FIG. 4.—Longitudinal study: COR . . . , a patient studied over 2·5 months, with a very low NCA level despite marked leucocytosis. She had a constant basophilia and excess of blasts in peripheral blood. N polymorphs = neutrophil polymorphs.

separated on Ficoll–Isopaque gradient. All these patients had an increased serum NCA level (77–458 ng/ml).

The mononuclear fraction (mainly immature granulocytic cells) often stained strongly. Myelocytes showed strong perinuclear staining. In one case, all the cells were strongly positive. In all the other cases, heterogeneous staining was seen, and immature cells that had apparently the same morphology were either strongly or weakly positive.

The staining of polymorphs was generally weaker than that of "mononuclear cells". Individual variation was striking, with sometimes 15% of negative cells. A granular pattern was often seen, and segmented polymorphs were less stained than band cells.

(5) *Determination of NCA cell content by radioimmunoassay*

NCA was assayed in the polymorph and the mononuclear fractions obtained from 17 CML patients.

Results are summarized in Table III, which shows that NCA content/$10^6$ cells is roughly the same in normal and leukaemic granulocytes. From the NCA content per $10^6$ cells, and the number of cells/$mm^3$, we established a new parameter—the NCA cellular content. The NCA values in all the maturing myeloid cells (1·1–18$\mu$g/ml) and all the polymorphs (2–20 $\mu$g/ml) were much higher than serum NCA level (66–458 ng/ml).

We examined the correlation between total cellular content and serum NCA level in these 17 CML patients. The correlation was better between serum level and maturing myeloid cell content ($r = 0{\cdot}448$), than between serum level and polymorph content ($r = 0{\cdot}118$), but the significance was weak even in the first comparison ($P = 0{\cdot}10$).

### DISCUSSION

The results described here for CML patients reinforce our previous conclusions. CML patients generally had higher serum NCA levels than normal subjects; however mean values were lower than those published by others (Wahren *et al.*, 1980).

TABLE II.—*Coefficient correlation (r) between serum NCA level and myeloid cell number*

| | NCA/total number of WBC | NCA/number of polymorphs | NCA/number of maturing myeloid cells[a] |
|---|---|---|---|
| Whole CML group | 0·567 ($P<0{\cdot}001$) | 0·327 ($P=0{\cdot}01$) | 0·586 ($P<0{\cdot}001$) |
| Patients with active disease | 0·298 (NS) | 0·068 (NS) | 0·413 ($P=0{\cdot}02$) |
| Patients with blast crisis | 0·354 (NS) | 0·507 ($P=0{\cdot}05$) | 0·594 ($P<0{\cdot}01$) |

[a] Maturing myeloid cells: promyelocytes + myelocytes + metamyelocytes.

TABLE III.—*Determination of NCA in cells from peripheral blood*

| | Number | Range (ng/$10^6$ cells) | Mean value (ng/ml) | s.d. |
|---|---|---|---|---|
| Normal subjects (polymorphs) | 11 | 29–99 | 62·07 | 21·39 |
| CML patients (polymorphs) | 17 | 27·5–105 | 67·54 | 22·81 |
| CML patients (mononuclear fraction) | 17 | 16–143 | 66·42 | 34·36 |

As it is easy to estimate the tumour mass according to the number of WBC per blood $mm^3$, CML might be one of the best models to study the parameters regulating the serum level of an antigen produced by tumour cells. This is the reason why we attempted to investigate the relationship between serum NCA concentration and WBC (or polymorphs) and, for some patients, to correlate serum NCA level with the NCA cell content of the granulocytic cells.

The relationship between serum NCA level and WBC count (polymorphs or maturing myeloid cells) was not generally strong in the whole CML population or in the two subsets, and no coefficient of correlation exceeded 0·6. However, the correlation was improved between serum NCA level and maturing myeloid cells counts—cells which appeared the more strongly stained in the immunoperoxidase studies. These results are rather different from those published by Wahren *et al.* (1982), who found a good correlation between serum NCA level and WBC number for the whole CML group.

Our data were the same from longitudinal studies. We have shown that the intra-subject correlation between serum NCA level and WBC (or polymorphs) count was better than the inter-subject correlation, since we found almost identical serum NCA levels in CML patients with very different WBC or polymorph counts. Patients studied during chemotherapy often revealed a parallelism between serum NCA level and WBC number; serum NCA level appears to reflect the response to treatment.

The immunocytological study of some individual patients in blast crisis permitted the conclusion that normal or low serum NCA level, despite a high WBC count, correlated with weak cell staining. This was especially true for patients in blast crisis: the same observation was made by Heuman *et al.* (1979). The results may be explained by the low synthesis of NCA in the immature leukaemic cells appearing in the blood.

Furthermore, we failed to demonstrate a correlation between serum NCA level and NCA content of granulocytic cells determined by radioimmunoassay in 17 untreated patients in active phase.

It is worthwhile stressing that the mean value of cellular NCA was the same for normal and CML granulocytic cells. This result is in accordance with those of Wahren *et al.* (1980). If we consider now the cell NCA content/ml of blood, values were much greater than the serum NCA levels. This means that only a small percentage of cellular NCA reaches the plasma. The correlation between total cell content and serum level of NCA is poor, this has to be seen in the light of the large variation of serum NCA concentration in CML patients having roughly similar WBC counts.

If these variations can at best be only partially explained by the NCA concentrations in myeloid cells, then other hypotheses have to be considered.

The first explanation is the possible contribution of other cells which are known to contain NCA such as alveolar macrophages (Burtin *et al.*, 1975), monocytes (Burtin & Fondanèche, 1981), or epithelial cells of the gastrointestinal mucosa (Burtin *et al.*, 1973). However, it is likely that in CML patients the main source of NCA is the granulocytic cells.

Another possible explanation lies in the rate of NCA release from cells and the rate of NCA catabolism. Here we have recourse to experience with CEA; for example the very high levels of serum CEA found in medullary carcinomas of the thyroid could be explained by the release of CEA from the basal pole of cancerous cells into the connective tissue, then into the peripheral blood (Burtin *et al.*, 1979). In contrast, breast tumours generally contain intracytoplasmic CEA (von Kleist, 1980), which fails to enter the blood, and serum CEA is normal or only weakly elevated. Thus high tissue concentrations of the same antigen may lead to very different serum levels, due to parameters such as the release rate. In common with NCA, the cellular amount of this antigen is several 100—fold higher than its serum level. Thus it is easy to understand how a small variation in the rate of NCA release may greatly influence the serum level of this antigen.

We have studied NCA release from the granulocytic cells of normal subjects and CML patients under non-physiological conditions *in vitro*. These appear to demonstrate that NCA release is slow in both categories, and extremely variable from one subject to another.

If this variability exists also *in vivo*, it could explain, at least in part, the intersubject variation of serum NCA levels reported herein.

We are grateful to Professor E. H. Cooper (Unit for Cancer Research, University of Leeds, England) for his kind and competent cooperation.

We thank Mrs Maunoury for her statistical analyses.

### REFERENCES

BORDES, M., KNOBEL, S. & MARTIN, F. (1975) Carcineoembryonic antigen (CEA) and related antigens in blood cells and haematopoietic tissues. *Eur. J. Cancer*, **12**, 783.

BURTIN, P., VON KLEIST, S., SABINE, M. C. & KING, M. (1973) Immunohistological localization of carcinoembryonic antigen and non-specific cross-reacting antigen in gastrointestinal normal and tumoral tissues. *Cancer Res.*, **33**, 3299.

BURTIN, P. & CHAVANEL, G. (1973) A new and fast method of preparation of CEA. *Ann. Immunol.*, **124**, 583.

BURTIN, P., QUAN, P. C. & SABINE, M. C. (1975) Non-specific cross-reacting antigen as marker for human polymorphs, macrophages and monocytes. *Nature*, **255**, 714.

BURTIN, P., CALMETTES, C. & FONDANECHE, M. C. (1979) CEA and non-specific cross-reacting antigen (NCA) in medullary carcinomas of the thyroid. *Int. J. Cancer*, **23**, 741.

BURTIN, P., FLANDRIN, G. & FONDANECHE, M. C. (1980) Presence of NCA (non-specific cross-reacting antigen) in the cells of the human granulocytic series. *Blood Cells*, **6**, 263.

BURTIN, P. & FONDANECHE, M. C. (1981) Characterization of the non-specific cross-reacting antigen (NCA) in human monocytes. *Clin. Immunol. Immunopathol.* **20**, 146.

DARCY, D., TUBERVILLE, C. & JAMES, R. (1973) Immunological study of carcinoembryonic antigen (CEA) and a related glycoprotein. *Br. J. Cancer*, **28**, 147.

EDGINGTON, T. S., PLOW, E. F., HERBERMAN, R. & 5 others (1976) A comparison of CEA-S and CEA concentrations in sera and the independence of CEA-S and blood group antigens. *Bull. Cancer*, **63**, 613.

EGAN, M. L., LAUTENSCHLEGER, J. T., COLIGAN, J. E. & TODD, G. W. (1972) Radioimmune assay of carcinoembryonic antigen. *Immunochemistry*, **9**, 289.

FRÉNOY, N. & BURTIN, P. (1980) Non specific cross-reacting antigen serum concentration in myeloid leukemias. *Clin. Chim. Acta*, **103**, 23.

GRAHAM, R. C., LUNDHOLM, V. & KARNOVSKI, M. J. (1965) Cytochemical demonstration of peroxidase activity with 3-amino-3-ethyl-carbazole. *J. Histochem. Cytochem.*, **13**, 150.

HEUMANN, D., CANDARDJIS, P., CARREL, S. & MACH, J. P. (1979) Identification of the normal glycoprotein (NGP) cross-reacting with CEA as differentiation antigen of myeloid cells and macrophages. In *Carcinoembryonic Proteins* (Ed. Lehmann)., Amsterdam: Elsevier-North Holland. p. 3.

HUNTER, W. M. & GREENWOOD, P. C. (1964) A radio-immunoelectrophoretic assay for human growth hormone. *Biochem. J.*, **91**, 43.

MACH, J. P. & PUSZTASZERI, G. (1972) Demonstration of a partial identity between CEA and a normal glycoprotein. *Immunochemistry*, **9**, 1031.

NEWMAN, E. S., PETRAS, E. S., HAMILTON, J. G., HAGER, H. J. & HANSEN, M. J. (1972) Demonstration of two tumor associated antigens in human colonic adenocarcinoma. *Fed. Proc.*, **31**, 639.

VON KLEIST, S., CHAVANEL, G. & BURTIN, P. (1972) Identification of a normal antigen that cross-reacts with the carcino-embryonic antigen. *Proc. Natl Acad. Sci.*, **69**, 2492.

VON KLEIST, S., TROUPEL, S., KING, M. & BURTIN, P. (1977*a*) A clinical comparison between non-specific cross-reacting antigen and CEA in patient's sera. *Br. J. Cancer*, **35**, 875.

von Kleist, S., Troupel, S., King, M. & Burtin, P. (1977*b*) Determination of levels of carcino-embryonic antigen and non-specific cross-reacting antigen in the sera of neonatal infants. *J. Natl Cancer Inst.*, **59**, 1621.

von Kleist, S. (1980) Communication to the 7th Congress of International Society for Oncodevelopmental Biology and Medicine, Tallin (U.S.S.R).

Wahren, B., Gahrton, G. & Hammarström, S. (1980) Non-specific cross-reacting antigen in normal and leukemic myeloid cells and serum of leukemic patients. *Cancer Res.*, **40**, 2039.

Wahren, B., Gahrton, G. Ruden , U. & Hammarström, S. (1982) Clinical evaluation of NCA in patients with chronic myelocytic leukemia. *Int. J. Cancer*, **29**, 133.